# Sex-dependent association of serum uric acid levels with amyloid accumulation among amyloid-positive older adults

**DOI:** 10.1371/journal.pone.0296738

**Published:** 2024-02-07

**Authors:** Guanan Zhao, Jingjing Guan, for the Alzheimer’s Disease Neuroimaging Initiative

**Affiliations:** 1 Department of Urology, Lishui City People’s Hospital, Zhejiang, China; 2 Department of Pharmacy, Lishui City People’s Hospital, Zhejiang, China; University of Catania Department of Surgical and Medical Sciences Advanced Technologies GF Ingrassia: Universita degli Studi di Catania Dipartimento di Scienze Mediche Chirurgiche e Tecnologie Avanzate GF Ingrassia, ITALY

## Abstract

We aimed to examine the potential effect of sex on the longitudinal association of baseline serum uric acid levels with brain amyloid accumulation over time among older adults with and without abnormal amyloid. At baseline, the study sample comprised 499 older adults, including 276 men and 223 women. Linear mixed-effects regression models were fitted to estimate the individual slopes of change in brain amyloid accumulation [as measured by AV45 standardized uptake value ratio (SUVR)] over time. At baseline, we did not observe a relationship between serum uric acid levels and brain amyloid deposition in women or men regardless of amyloid status. Among amyloid negative subjects, women and men did not differ in the relationship between baseline serum uric acid and the annual change in amyloid accumulation in subjects with normal amyloid levels. In amyloid positive women, serum uric acid levels were not associated with the annual change in amyloid accumulation (unstandardized β = 0.0005, SE = 0.0006, p value = 0.4179). However, in amyloid positive men, serum uric acid levels were negatively associated with the annual change in amyloid accumulation (unstandardized β = -0.0015, SE = 0.0005, p value = 0.0048). These findings support a potential sex-specific effect on the relationship between serum uric acid levels and amyloid accumulation among amyloid positive older adults.

## Introduction

Uric acid is a naturally produced antioxidant that accounts for nearly 60% of the antioxidant capacity in humans. A growing body of studies has investigated the relationship between uric acid and cognitive decline or Alzheimer’s disease (AD) and yielded mixed results [1–6]. Several studies have examined the association of uric acid with AD from the neuropathological perspective, with contradictory findings. For example, a positive association between serum uric acid levels and CSF amyloid beta (Aβ)_42_ was found in cognitively healthy men (β = 0.55, P = .04) [7]. Li and colleagues reported that serum uric acid levels were negatively correlated with CSF Aβ_42_ levels among cognitively healthy older adults [e.i., individuals with highest uric acid (> 75% percentile) had lower CSF Aβ_42_ levels relative to those with lowest uric acid (β = –5.1×10^−4^, p = 0.019)] [8]. However, linear regression models adjusted for several potential covariates suggested that there was no relationship between serum uric acid levels and brain Aβ deposition (as measured by [^11^C] Pittsburgh compound B (PiB)-positron emission tomography (PET)) in non-demented Korean participants (β = 0.001,95% CI = −0.015 to 0.017, p = 0.877) [9].

Given that previous studies on the relationship between uric acid and amyloid deposition were cross-sectional in design and yielded inconsistent results, and that no study has examined the potential moderating effect of sex on the relationship between serum uric acid levels and amyloid accumulation, we aimed to investigate whether there are sex differences in the relationship between uric acid and the slopes of amyloid accumulation among older adults with and without abnormal amyloid.

## Materials and methods

### Alzheimer’s Disease Neuroimaging Initiative (ADNI) database

Data used in the present study were extracted from the ADNI study (https://adni.loni.usc.edu/), which is an ongoing prospective study using numerous methods, such as neuropsychological and neuroimaging markers, to measure the clinical progression of mild cognitive impairment (MCI) and early AD. The Institutional Review Board of each participating study site approved the ADNI study and all ADNI participants provided written consent.

### Participants

Given the longitudinal design of this analysis, we included older adults with serum uric acid data available at baseline and at least two [^18^F] Florbetapir PET (FBP-PET) scans (for measurement of brain amyloid load). At baseline, 499 older adults were consisting of 147 cognitively normal older adults, 327 older adults with MCI and 25 patients with mild AD dementia. A participant was classified as having normal cognition if he/she had a Mini-Mental Status Examination (MMSE) [10] score between 24 and 30 and a Clinical Dementia Rating (CDR) [11] score of 0. A participant was diagnosed with MCI if he/she had an MMSE score between 24 and 30, scored 0.5 on the CDR, showed objective memory impairment as evidenced by Wechsler Memory Scale Logical Memory II, and did not have a diagnosis of dementia. A subject was diagnosed with mild AD dementia if he/she had memory complaint, an MMSE score between 20–26, a global CDR score of 0.5 or 1, memory impairment indicated by the Logical Memory II subscale from the Wechsler Memory Scale-Revised and met the NINCDS/ADRDA criteria for probable AD dementia [12]. The authors cannot have access to information that could identify individual participants during or after data collection.

### Uric acid data

Uric acid data were obtained from the Biospecimen data section of the ADNI database. The detailed protocols for the collection and measurement of serum uric acid levels can be found in the Procedures Manual of the ADNI study (https://adni.loni.usc.edu/wp-content/uploads/2008/07/adni2-procedures-manual.pdf).

### Determination of amyloid load

Participants’ amyloid accumulation data were extracted from the ADNI data file “ADNIMERGE.csv”. Amyloid accumulation was determined by PET imaging with ^18^F-AV-45 (florbetapir). The summarized standardized uptake value ratios (SUVRs) normalized to the cerebellum were extracted from the ADNI database. Further detailed procedures of data collection and processing can be found at the website: https://adni.loni.usc.edu/wp-content/uploads/2010/05/ADNI2_PET_Tech_Manual_0142011.pdf. A participant was regarded as amyloid positive if AV45 SUVR > 1.1 based on a previous study [13].

### Statistical analysis

Baseline sample characteristics and clinical variables were compared between gender using Welch two-sample t-tests for continuous variables and Pearson’s Chi-squared tests for categorical variables. At baseline, Pearson’s correlation tests were applied to examine the relationships between serum uric acid levels and brain amyloid burden (as measured by AV45 SUVR) in two gender groups separately. In the longitudinal analyses, a linear mixed-effects regression model with AV45 data from multiple timepoints as the dependent variable and time since baseline (years) as the independent variable was fitted, and individual slopes of amyloid accumulation were extracted from the model. This model included a random intercept and a random slope for each participant. Subsequently, two linear regression models treating the slopes as the dependent variable and baseline serum uric acid levels as the independent variable were fitted separately in amyloid negative and amyloid positive participants. To examine the potential moderation effect of sex on the association of serum uric acid levels with the slopes of amyloid accumulation, the interaction term (uric acid*sex) was included in each model. These two models were adjusted for the effects of several covariates, including age, educational levels, APOE4 genotype, MMSE, and cognitive diagnosis. A p value < 0.05 was regarded as statistical significance. All statistical work was conducted in R statistical software, Version 4.1.2 [14].

## Results

### Sample characteristics by gender

The study sample comprised 499 participants including 276 men and 223 women (Table 1). Women were younger, less educated, and had lower serum uric acid levels than men (all p < 0.05). There was no difference in the percentage of APOE4 carriers, MMSE score, clinical diagnosis or amyloid PET SUVR between women and men (all p > 0.05). Additionally, patient characteristics stratified based on amyloid status and gender are shown in the S1 Table in S1 File.

**Table 1:** Sample characteristics by gender.

Characteristic	Overall, N = 499^1^	Male, N = 276^1^	Female, N = 223^1^	p-value^2^
Age, years	72 (7)	73 (7)	71 (7)	0.022
Education, years	16 (3)	17 (3)	16 (2)	<0.001
APOE4 status				>0.9
APOE4 noncarriers	282 (57%)	156 (57%)	126 (57%)	
APOE4 carriers	217 (43%)	120 (43%)	97 (43%)	
Clinical diagnosis				0.6
CN	147 (29%)	79 (29%)	68 (30%)	
MCI	327 (66%)	181 (66%)	146 (65%)	
AD	25 (5.0%)	16 (5.8%)	9 (4.0%)	
MMSE	28 (2)	28 (2)	28 (2)	0.3
Amyloid PET SUVR	1.18 (0.22)	1.18 (0.22)	1.19 (0.22)	0.6
Serum uric acid, mg/dL	5.47 (1.33)	5.97 (1.16)	4.84 (1.26)	<0.001

^1^Mean (SD); n (%)

^2^Welch Two Sample t-test; Pearson’s Chi-squared test

Abbreviation: MMSE: Mini Mental Status Examination; SUVR: standardized uptake value ratio.

### Cross-sectional relationship between serum uric acid levels and amyloid PET SUVR

**Fig 1:**
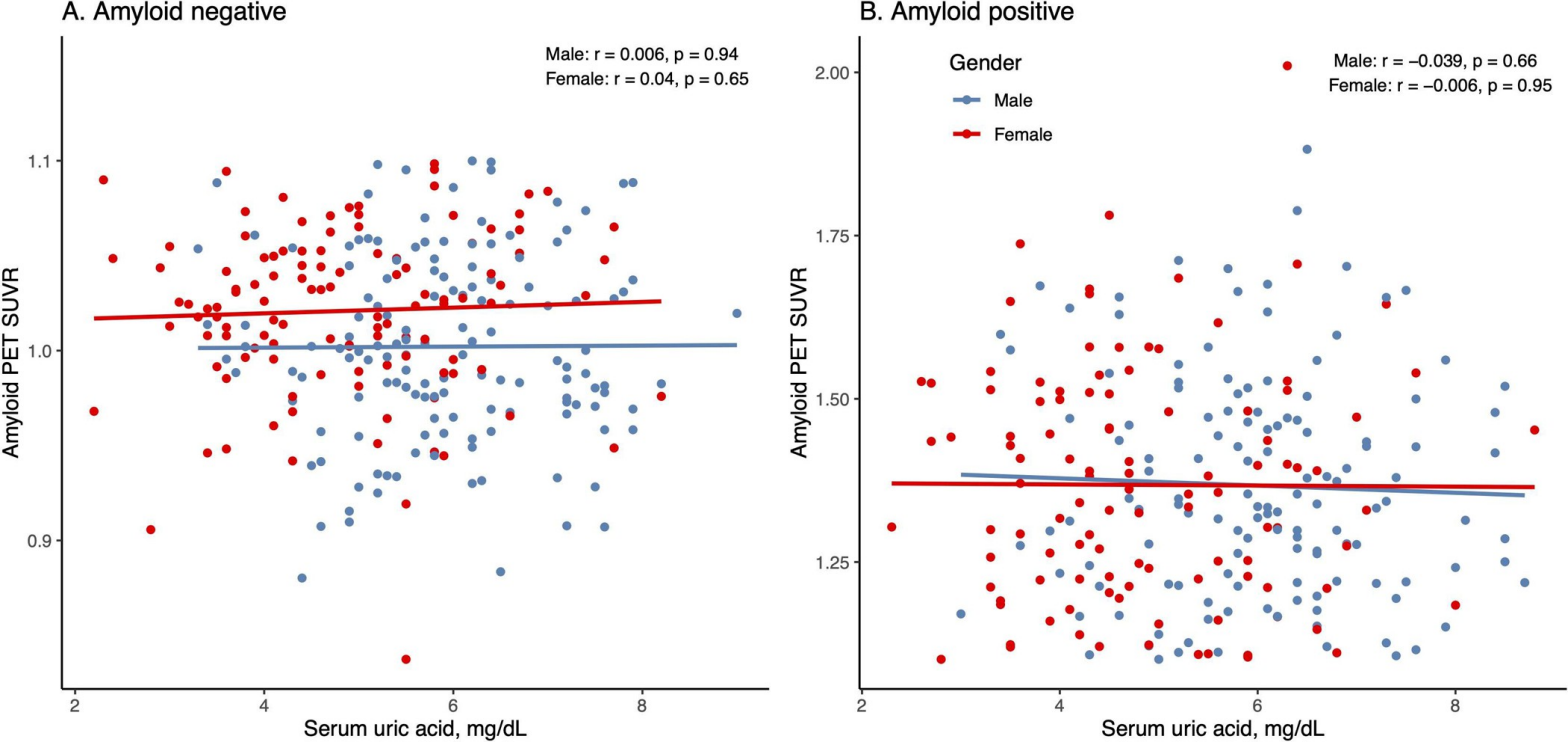
The relationship between serum uric acid levels and amyloid PET SUVR in women and men based on amyloid status. At baseline, serum uric acid levels were not associated with amyloid PET SUVR in women or men irrespective of amyloid status. Abbreviation: SUVR: standardized uptake value ratio.

Pearson’s correlation tests were used to examine the relationship between serum uric acid levels and amyloid PET SUVR in women and men at baseline. As shown in Fig 1, among amyloid-negative subjects, serum uric acid levels were not associated with amyloid PET SUVR in women (r = 0.04, p = 0.65) or in men (r = -0.006, p = 0.94). Among amyloid-positive subjects, serum uric acid levels were not associated with amyloid PET SUVR in women (r = -0.006, p = 0.95) or in men (r = -0.039, p = 0.66).

### The effect of sex on the association of serum uric acid levels with the slopes of amyloid accumulation

**Fig 2:**
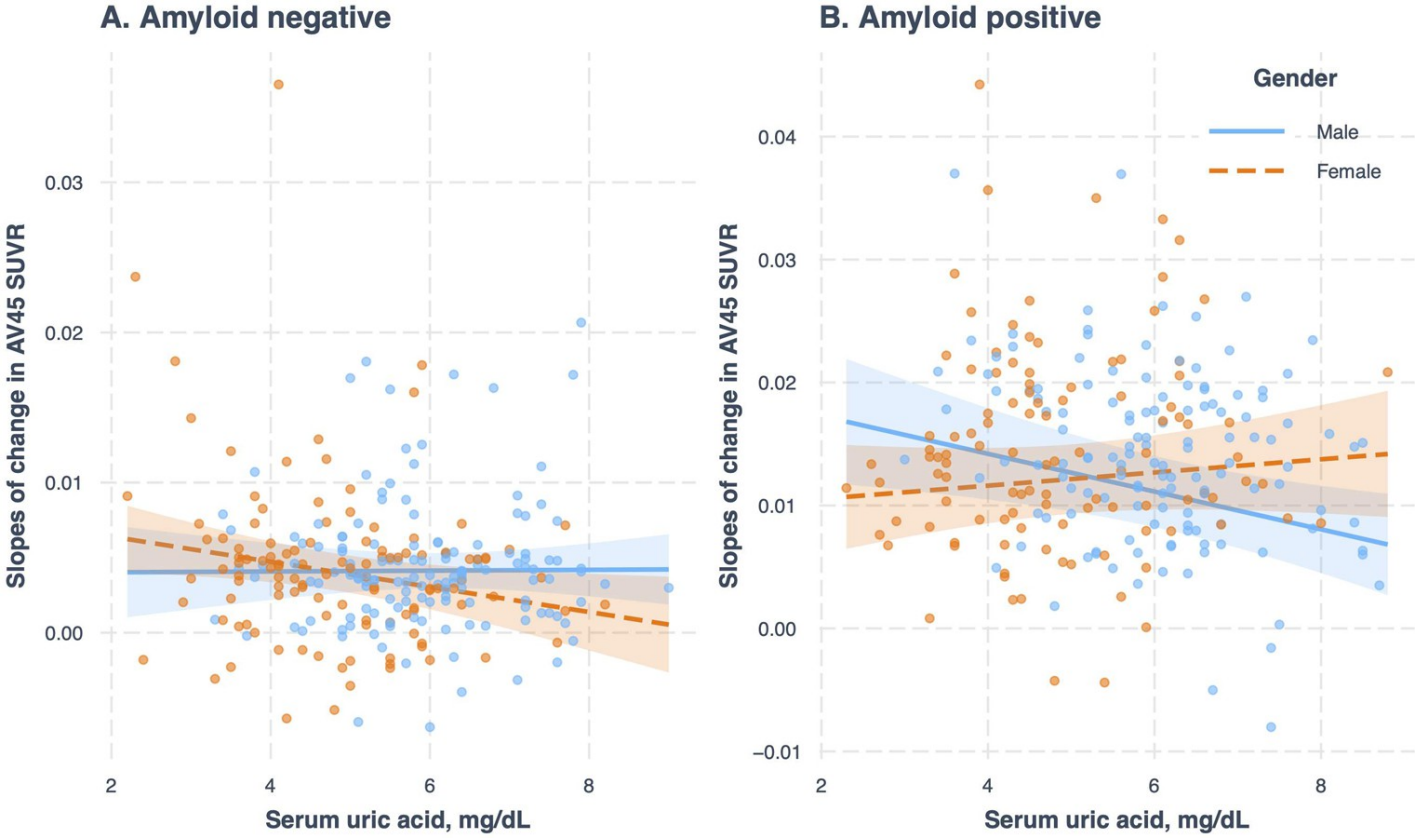
The effect of sex on the associations of baseline serum uric acid levels with the slopes of change in brain amyloid accumulation in amyloid-negative and amyloid-positive older adults.

Among amyloid negative and amyloid positive subjects, two linear regression models with the slopes of amyloid accumulation as the outcome and the interaction term (Uric acid × Gender) as the predictor were performed to examine the potential effect of gender on the relationship between uric acid and the slopes of amyloid accumulation (Table 2 and Fig 2). Among amyloid negative subjects, the interaction term (Uric acid × Female gender) was not significant (Coefficient = -0.0009, SE = 0.0005, p = 0.09), suggesting that women and men did not differ in the relationship between baseline serum uric acid and the annual change in amyloid accumulation in subjects with normal amyloid levels (Table 2 and Fig 2A). However, among amyloid positive subjects, the interaction term (Uric acid × Female gender) was significant (Coefficient = 0.0021, SE = 0.0008, p = 0.01), indicating that women and men differed in the relationship between baseline serum uric acid and the annual change in amyloid accumulation in subjects with abnormal amyloid levels (Table 2 and Fig 2B). To better understand these interactions among amyloid positive subjects, we further performed two separate linear regression models adjusting for several potential covariates (i.e., age, education, APOE4 status, MMSE, and clinical diagnosis) for women and men. In amyloid positive women, serum uric acid levels were not associated with the annual change in amyloid accumulation (unstandardized β = 0.0005, SE = 0.0006, p value = 0.4179). However, in amyloid positive men, serum uric acid levels were negatively associated with the annual change in amyloid accumulation (unstandardized β = -0.0015, SE = 0.0005, p value = 0.0048). Further, we also recoded the gender variable with female as the reference group, and the S2 Table in S1 File shows the summary of regression models.

**Table 2:** Summary of regression models by amyloid status.

	Amyloid negative			Amyloid positive		
Predictors	Coefficient	SE	p	Coefficient	SE	p
Age	0.00002	0.00004	0.65	0.00006	0.00008	0.44
Education	-0.000007	0.0001	0.96	0.0002	0.0002	0.27
APOE4 status (APOE4 carrier)	0.003	0.0007	< 0.001	0.0018	0.0011	0.09
MMSE	-0.0001	0.0002	0.67	0.0003	0.0003	0.31
Clinical diagnosis (MCI)	-0.0007	0.0007	0.26	0.0016	0.0014	0.25
Clinical diagnosis (AD)	-0.002	0.003	0.38	0.0038	0.0028	0.17
Uric acid	0.00003	0.0004	0.94	-0.0015	0.0006	0.006
Female gender	0.004	0.003	0.14	-0.01	0.004	0.015
Uric acid × Female gender	-0.0009	0.0005	0.09	0.0021	0.0008	0.01

Abbreviation: MMSE: Mini Mental Status Examination; MCI: Mild cognitive impairment; AD: Alzheimer’s disease. Notes: Coefficients are unstandardized β.

## Discussion

In this study, at baseline we did not observe a relationship between serum uric acid levels and brain amyloid deposition in women or men regardless of amyloid status. Among amyloid negative subjects, women and men did not differ in the relationship between baseline serum uric acid and the annual change in amyloid accumulation in subjects with normal amyloid levels. In amyloid positive women, serum uric acid levels were not associated with the annual change in amyloid accumulation. However, in amyloid positive men, serum uric acid levels were negatively associated with the annual change in amyloid accumulation. Our findings support a potential sex-specific effect on the association of serum uric acid levels with amyloid accumulation among amyloid positive older adults.

The finding that serum uric acid levels were not correlated with amyloid deposition in the cross-sectional analyses is consistent with a previous study [9], which showed that serum uric acid levels were not associated with cerebral amyloid accumulation (as measured by [^11^C] PiB-PET) in non-demented Korean participants (age range = 55–90 years). However, two previous studies examining the relationship between serum uric acid levels and CSF Aβ42 found contradictory results, with one showing a positive relationship (mean age = 70.9, standard deviation = 0.35) [7] and another negative (median age = 63, Interquartile range = 14) [8]. This inconsistency may be attributed to several factors, such as the cognitive status of the study sample (cognitively healthy vs older adults with different degrees of cognitive impairment), the mean age of the study sample, and methods utilized to measure Aβ levels [cerebral Aβ deposition (as measured by PET imaging) vs CSF Aβ42].

We found that women and men did not differ in the relationship between baseline serum uric acid and the annual change in amyloid accumulation in subjects with normal amyloid levels. However, among amyloid positive subjects, women and men differed in the relationship between baseline serum uric acid and the annual change in amyloid accumulation. To our knowledge, this is the first report of a sex-specific effect on the association of serum uric acid levels with the slopes of change in brain amyloid accumulation over time among amyloid positive older adults. In amyloid positive women, serum uric acid levels were not associated with the annual change in amyloid accumulation. However, in amyloid positive men, serum uric acid levels were negatively associated with the annual change in amyloid accumulation (unstandardized β = -0.0015, SE = 0.0005, p value = 0.0048). These findings are consistent with a previous longitudinal study, which demonstrated that among dementia-free subjects, baseline higher serum uric acid levels were associated with a slower decline in several cognitive domains in men, but not in women [15]. Emerging evidence has shown that uric acid plays a protective role against neurodegenerative processes by decreasing free radicals and oxidative stress [16, 17], although higher levels of uric acid are associated with a variety of cardiovascular risk factors and diseases [18], which may contribute to faster amyloid deposition and higher AD dementia incidence [19, 20].

This study should be interpreted by considering several limitations. First, given the observational nature of our study, we cannot clarify the causal relationship between uric acid and amyloid accumulation. Second, the ADNI participants represent a convenience sample. Thus, further population-based studies are needed to test the robustness of our findings when applying our results to other populations. Third, the sample size of the study is relatively small. Further studies with larger sample sizes are warranted to test the robustness of current findings. Fourth, while a significant interaction effect (uric acid*gender) was detected in amyloid-positive older adults, the actual clinical implications are not certain.

In conclusion, this study found a sex-specific association of serum uric acid levels with brain amyloid accumulation over time among amyloid positive older adults.

## Supporting information

S1 File Contains supporting tables. (DOCX)

## References

[1] Scheepers L, Jacobsson LTH, Kern S, Johansson L, Dehlin M, Skoog I. Urate and risk of Alzheimer’s disease and vascular dementia: A population-based study. Alzheimers Dement. 2019;15(6):754–63. Epub 2019/05/06. doi: 10.1016/j.jalz.2019.01.014 . 31056343

[2] Du N, Xu D, Hou X, Song X, Liu C, Chen Y, et al. Inverse Association Between Serum Uric Acid Levels and Alzheimer’s Disease Risk. Mol Neurobiol. 2016;53(4):2594–9. Epub 2015/06/19. doi: 10.1007/s12035-015-9271-6 . 26084440

[3] Latourte A, Soumaré A, Bardin T, Perez-Ruiz F, Debette S, Richette P. Uric acid and incident dementia over 12 years of follow-up: a population-based cohort study. Ann Rheum Dis. 2018;77(3):328–35. Epub 2017/07/30. doi: 10.1136/annrheumdis-2016-210767 . 28754803

[4] Beydoun MA, Canas JA, Dore GA, Beydoun HA, Rostant OS, Fanelli-Kuczmarski MT, et al. Serum Uric Acid and Its Association with Longitudinal Cognitive Change Among Urban Adults. J Alzheimers Dis. 2016;52(4):1415–30. Epub 2016/04/23. doi: 10.3233/JAD-160028 ; PubMed Central PMCID: PMC4902772. 27104899 PMC4902772

[5] Euser SM, Hofman A, Westendorp RG, Breteler MM. Serum uric acid and cognitive function and dementia. Brain. 2009;132(Pt 2):377–82. Epub 2008/11/28. doi: 10.1093/brain/awn316 . 19036766

[6] Verhaaren BF, Vernooij MW, Dehghan A, Vrooman HA, de Boer R, Hofman A, et al. The relation of uric acid to brain atrophy and cognition: the Rotterdam Scan Study. Neuroepidemiology. 2013;41(1):29–34. Epub 2013/04/04. doi: 10.1159/000346606 . 23548762

[7] Fatima T, Jacobsson LTH, Kern S, Zettergren A, Blennow K, Zetterberg H, et al. Association between serum urate and CSF markers of Alzheimer’s disease pathology in a population-based sample of 70-year-olds. Alzheimers Dement (Amst). 2021;13(1):e12241. Epub 2021/12/23. doi: 10.1002/dad2.12241 ; PubMed Central PMCID: PMC8652407. 34934798 PMC8652407

[8] Li L-L, Ma YH, Bi Y-l, Sun F-R, Hu H, Hou X-H, et al. Serum Uric Acid May Aggravate Alzheimer’s Disease Risk by Affecting Amyloidosis in Cognitively Intact Older Adults: The CABLE Study. Journal of Alzheimer’s disease: JAD. 2021;81(1):389–401. Epub 2021/04/06. doi: 10.3233/JAD-201192 . 33814427

[9] Kim JW, Byun MS, Yi D, Lee JH, Jeon SY, Ko K, et al. Serum Uric Acid, Alzheimer-Related Brain Changes, and Cognitive Impairment. Front Aging Neurosci. 2020;12:160. Epub 2020/06/26. doi: 10.3389/fnagi.2020.00160 ; PubMed Central PMCID: PMC7291838. 32581770 PMC7291838

[10] Folstein MF, Folstein SE, McHugh PR. "Mini-mental state". A practical method for grading the cognitive state of patients for the clinician. J Psychiatr Res. 1975;12(3):189–98. Epub 1975/11/01. doi: 10.1016/0022-3956(75)90026-6 . 1202204

[11] Morris JC. The Clinical Dementia Rating (CDR): current version and scoring rules. Neurology. 1993;43(11):2412–4. Epub 1993/11/01. doi: 10.1212/wnl.43.11.2412-a . 8232972

[12] Mckhann G, Drachman DA, Folstein MF, Katzman R, Price DL, Stadlan EM. Clinical diagnosis of Alzheimer’s disease. Neurology. 1984;34:939 -. 6610841 10.1212/wnl.34.7.939

[13] Landau SM, Breault C, Joshi AD, Pontecorvo MJ, Mathis CA, Jagust WJ, et al. Amyloid-β Imaging with Pittsburgh Compound B and Florbetapir: Comparing Radiotracers and Quantification Methods. The Journal of Nuclear Medicine. 2013;54:70–7. 23166389 10.2967/jnumed.112.109009 PMC3747730

[14] Team RC. R: A language and environment for statistical computing. MSOR connections. 2014;1.

[15] Kueider AM, An Y, Tanaka T, Kitner-Triolo MH, Studenski S, Ferrucci L, et al. Sex-Dependent Associations of Serum Uric Acid with Brain Function During Aging. J Alzheimers Dis. 2017;60(2):699–706. Epub 2017/09/19. doi: 10.3233/JAD-170287 ; PubMed Central PMCID: PMC6112110. 28922153 PMC6112110

[16] Squadrito GL, Cueto R, Splenser AE, Valavanidis A, Zhang H, Uppu RM, et al. Reaction of uric acid with peroxynitrite and implications for the mechanism of neuroprotection by uric acid. Arch Biochem Biophys. 2000;376(2):333–7. Epub 2000/04/25. doi: 10.1006/abbi.2000.1721 . 10775420

[17] Kim TS, Pae CU, Yoon SJ, Jang WY, Lee NJ, Kim JJ, et al. Decreased plasma antioxidants in patients with Alzheimer’s disease. Int J Geriatr Psychiatry. 2006;21(4):344–8. Epub 2006/03/15. doi: 10.1002/gps.1469 . 16534775

[18] Saito Y, Tanaka A, Node K, Kobayashi Y. Uric acid and cardiovascular disease: A clinical review. J Cardiol. 2021;78(1):51–7. Epub 2021/01/04. doi: 10.1016/j.jjcc.2020.12.013 . 33388217

[19] de Bruijn RF, Ikram MA. Cardiovascular risk factors and future risk of Alzheimer’s disease. BMC Med. 2014;12:130. Epub 2014/11/12. doi: 10.1186/s12916-014-0130-5 ; PubMed Central PMCID: PMC4226863. 25385322 PMC4226863

[20] Gottesman RF, Schneider AL, Zhou Y, Coresh J, Green E, Gupta N, et al. Association Between Midlife Vascular Risk Factors and Estimated Brain Amyloid Deposition. Jama. 2017;317(14):1443–50. Epub 2017/04/12. doi: 10.1001/jama.2017.3090 ; PubMed Central PMCID: PMC5921896. 28399252 PMC5921896

