# Supplementary material for: Sex-dependent association of serum uric acid levels with amyloid accumulation among amyloid-positive older adults
Source: PLoS One. 2024 Feb 7;19(2):e0296738. doi: 10.1371/journal.pone.0296738 (PMC10849271; doi:10.1371/journal.pone.0296738)
Supplement: S1 File — (DOCX) [file pone.0296738.s001.docx]

| **Characteristic** | **Amyloid- Males**  N = 143^1^ | **Amyloid+ Males**  N = 133^1^ | **Amyloid- Females**  N = 115^1^ | **Amyloid+ Females**  N = 108^1^ |
| --- | --- | --- | --- | --- |
| Age, years | 71 (7) | 74 (6) | 70 (7) | 72 (7) |
| Education, years | 17 (2) | 17 (3) | 16 (2) | 15 (2) |
| APOE4 status |  |  |  |  |
| APOE4 noncarriers | 111 (78%) | 45 (34%) | 89 (77%) | 37 (34%) |
| APOE4 carriers | 32 (22%) | 88 (66%) | 26 (23%) | 71 (66%) |
| Clinical diagnosis |  |  |  |  |
| CN | 58 (41%) | 21 (16%) | 43 (37%) | 25 (23%) |
| MCI | 81 (57%) | 100 (75%) | 72 (63%) | 74 (69%) |
| AD | 4 (2.8%) | 12 (9.0%) | 0 (0%) | 9 (8.3%) |
| MMSE | 29 (2) | 27 (2) | 29 (1) | 28 (2) |
| Amyloid PET SUVR | 1.00 (0.05) | 1.37 (0.17) | 1.02 (0.04) | 1.37 (0.18) |
| Serum uric acid, mg/dL | 5.93 (1.13) | 6.01 (1.19) | 4.85 (1.25) | 4.83 (1.28) |
| ^1^Mean (SD); n (%); Abbreviation: MMSE: Mini Mental Status Examination; SUVR: standardized uptake value ratio. | | | | |

**Table S1. Sample characteristics by amyloid status and gender**

**Table S2. Summary of regression models by amyloid status (gender variable was coded with female as reference)**

|  |  | Amyloid negative | | |  | Amyloid positive | | |
| --- | --- | --- | --- | --- | --- | --- | --- | --- |
| Predictors |  | Coefficient | SE | p |  | Coefficient | SE | p |
| Age |  | 0.00002 | 0.00004 | 0.65 |  | 0.00006 | 0.00008 | 0.44 |
| Education |  | -0.000007 | 0.0001 | 0.96 |  | 0.0002 | 0.0002 | 0.27 |
| APOE4 status (APOE4 carrier) |  | 0.003 | 0.0007 | < 0.001 |  | 0.0018 | 0.0011 | 0.09 |
| MMSE |  | -0.0001 | 0.0002 | 0.67 |  | 0.0003 | 0.0003 | 0.31 |
| Clinical diagnosis (MCI) |  | -0.0007 | 0.0007 | 0.26 |  | 0.0016 | 0.0014 | 0.25 |
| Clinical diagnosis (AD) |  | -0.002 | 0.003 | 0.38 |  | 0.0038 | 0.0028 | 0.17 |
| Uric acid |  | -0.0008 | 0.0004 | 0.02 |  | 0.0005 | 0.0006 | 0.36 |
| Male gender |  | -0.004 | 0.003 | 0.14 |  | 0.01 | 0.004 | 0.015 |
| Uric acid × Male gender |  | 0.0009 | 0.0005 | 0.09 |  | -0.0021 | 0.0008 | 0.01 |

Abbreviation: MMSE: Mini Mental Status Examination; MCI: Mild cognitive impairment; AD: Alzheimer’s disease. Notes: Coefficients are unstandardized β.
